# Magnitude, trends and prevention of road traffic accidents in the Republic of South Africa

**DOI:** 10.4102/safp.v62i1.5032

**Published:** 2020-05-26

**Authors:** Adeloye A. Adeniji, Langalibalele H. Mabuza, Elton Titus

**Affiliations:** 2 Department of Family Medicine, Stellenbosch University, Cape Town, South Africa; 2 Ceres Hospital, Ceres, Cape Winelands District, Ceres, Western Cape, South Africa; 3 Department of Family Medicine and Primary Primary Health Care, Sefako Makgatho Health Sciences University, Pretoria, South Africa; 4 Eastern Cape Provincial Government, King Williams Town, South Africa

**Keywords:** road traffic injury (RTI), World Health Organization (WHO), South Africa (SA), low income, middle income, high income, gross domestic product (GDP)

## Abstract

Road traffic injuries (RTIs) constitute one of the five major disease burdens in South Africa with high mortality and morbidity. Thus far, the scientific enquiry into this burden has not been accompanied by successful government efforts to meet the challenge. Currently, more than 1.2 million people die and 20–50 million are with disabilities annually country-wide from RTIs. While there is a progressive reduction in mortality related to human immunodeficiency virus (HIV) conditions as a result of interventions, the mortality from RTI is seen to be progressively worsening as a result of increasing motorisation. There are disparities in the burden of RTI across different countries, with low- and middle-income countries bearing the highest burden. In Africa, 24.1 per 100 000 people die annually from RTI compared to 10.3 per 100 000 people in European countries. This opinion article investigates the magnitude, trends and prevention of RTI in South Africa.

## Introduction

**FIGURE 1:**
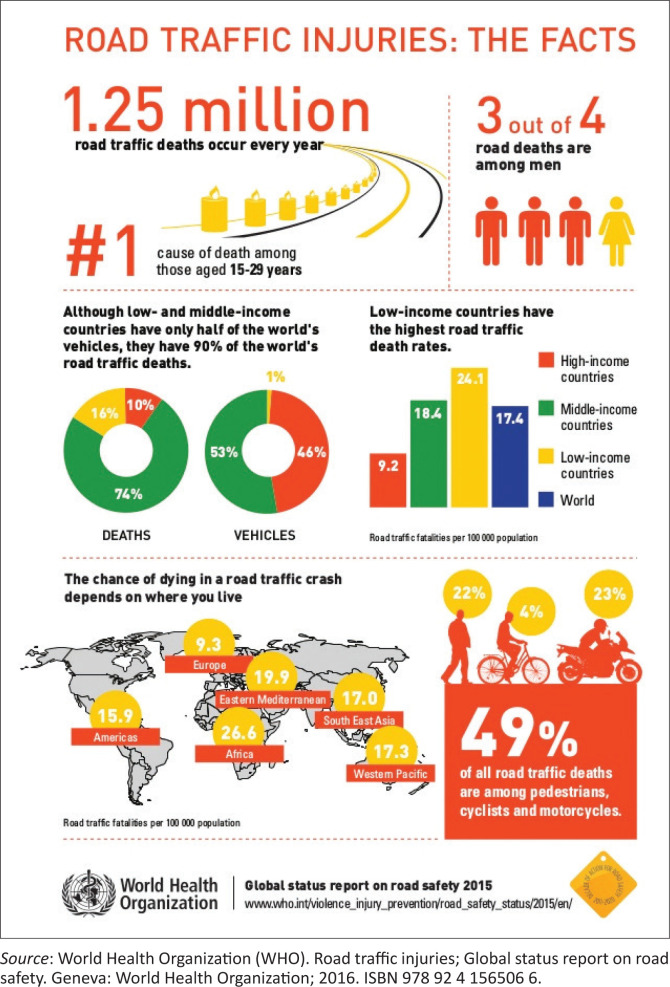
Infographics on road traffic injuries.

Globally, road traffic injuries (RTIs) claim more than 1.2 million lives each year (Figure 1) with a huge impact on health and development. It is the leading cause of death among young people aged between 15 and 29 years.^1^ It has been estimated that over the first 30 years of the 21st century, more cars will be produced than in the first 100 years of motorisation. The bulk of these vehicles will be introduced to the roads of low- and middle-income countries (LMICs), like South Africa (SA). This implies that the situation regarding RTIs will worsen, given the high prevalence these countries are already experiencing.^2^ It has been reported that globally, 90% of road traffic deaths occur in the LMICs.^1^

The widely accepted decade of advocacy for global road safety (2000–2010) and the decade of global action for road safety (2011–2020)^1^ seem to have had minimal or no impact on the level of RTIs in low- and middle-income economies, particularly in sub-Saharan Africa.

In SA, ‘Injuries’ is a component of the quadruple disease burden, and RTIs ranked second to interpersonal violence among the injury-related mortalities.^3^ The high number of accidents on South African roads does not only result in loss of human life leading to pain, grief and suffering but has also a negative effect on the well-being of South Africans and on the socio-economic development of the country.^4^ The total cost of South African road traffic accidents in 2015 was estimated at R142.95 billion, which equated to 3.4% of her gross domestic product (GDP).^4^

Road traffic injuries have a substantial impact on both household income and the national economy. A family affected by RTIs may be subjected to prolonged medical care, funeral costs and loss of income as a result of the disability or death of the breadwinner.^5^ In his principles of Family Medicine, McWinney stated that a clinician should view any encounter with a patient as an opportunity for health promotion and prevention, the clinician should seriously consider the context of a presenting patient and not only focus on their disease and also that the clinician is effective to the extent that he or she functions within a network of other professionals rather than as an island.^6^ These principles are relevant from a family physician’s perspective in addressing the problem of RTIs.

## Decade of action for road safety

**FIGURE 2:**
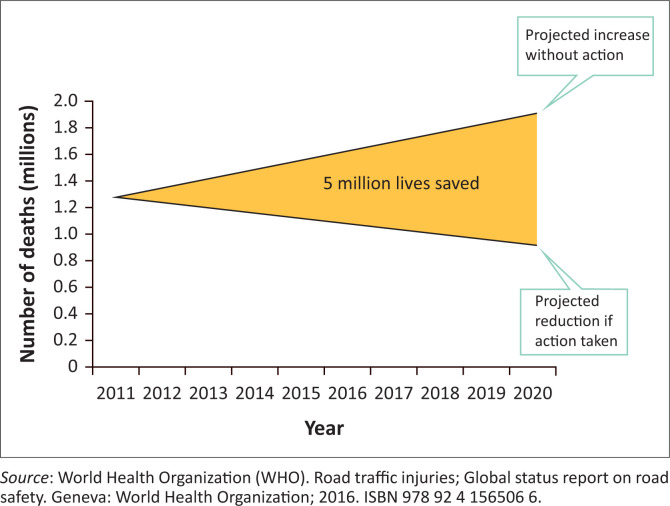
Projection 2011–2020.

A Global Plan has been put in place to support the implementation of The World Report recommendations aimed at stabilising and reducing the level of road traffic fatalities in LMICs by 2020.^3^ The aim is to reduce the death toll and serious injuries from RTIs by 50% by 2020, which will represent 5 million lives saved and 50 million serious injuries prevented. This will translate to a social benefit of more than $3 trillion^3^ (Figure 2).

## Risk factors for road traffic injuries in South Africa

While the attributable risk factors of RTI have been clearly identified by some studies in other parts of the world, efforts directed at achieving the same in SA have yielded minimal or no encouraging results.

### Behavioural risk factors

In 2019, the ‘Arrive Alive’ campaign was established to meet the challenges of RTIs in SA. A total of 9414 motorists were arrested for various offences during the festive season. These included drunken driving, speeding, reckless, outstanding warrants of arrest and negligent driving.^7^ Substance abuse, aggressive driving, fatigue, distractions (e.g. use of cell phone while driving) and poor safety habits (e.g. not using seatbelts) were found to be common causes of RTIs. Alcohol abuse has also been found to contribute significantly to RTIs.^8^ The South African blood alcohol concentration (BAC) limit (0.05 g/dL) becomes exceeded in men (mass < 80 kg) after taking more than two standard drinks and after more than one standard drink in women (mass < 55 kg).^8^ The limit for professional drivers is 0.02 g/dL.^8^

### Societal risk factors

These include a culture of impunity (lawlessness as a result of ineffective enforcement, particularly for minibus taxi and luxury vehicle drivers).^8^ Truck drivers and other heavy vehicle drivers have also been found to break traffic rules. Law enforcement personnel were among the people apprehended for bribery and other lawless behaviour during the 2019 campaign aimed at reducing RTI in SA. A figure of 58 699 fines were issued for over-speeding during the festive season of 2019, and a driver was arrested in January 2020 for travelling at a speed of 300 km/h.^7^

### Biological risk factors

Drivers aged between 15 and 29 years have been found to be at a higher risk of RTIs, with male drivers more prone than their female counterpart.^1^ Of Cape Town traffic deaths, 78% were related male population.^8^ The young drivers tend to take more risks, some as a result of peer pressure. Poor hearing, vision and attention also constitute important biological risk factors of RTI.^8^

### Structural risk factors

Structural risk factors include a combination of environmental and vehicular factors. Road maintenance, markings and visibilities are important structural considerations in road safety. Vehicle size, weight, speed, momentum and the gasoline tank that can explode on impact make cars dangerous machines.^9^ Maintenance as well as roadworthiness of these machines constitute important safety indices. Over the 2019 December festive period, 6358 unroad-worthy vehicles were discontinued and 3814 impounded in SA.^7^ Whether the re-invention of electric vehicles will worsen the incidence of RTI fatalities in SA is still to be established.^9^ However, there may be an increase in RTI with associated electrocution and electrical burns.

## Trends of road traffic injuries in South Africa

**FIGURE 3:**
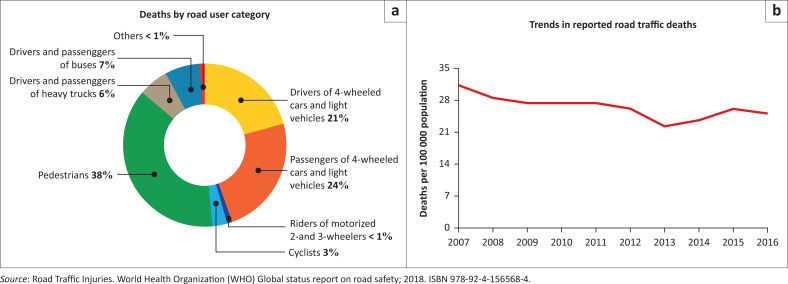
Trends in road traffic deaths in the Republic of South Africa 2007–2016.

In 2016, pedestrians constituted the highest number of victims of road traffic death in SA (38%),^1^ while pedestrians and drivers of four-wheeled cars and light vehicles constituted more than 50% of road traffic deaths (Figure 3a).^1^ In comparison, pedestrian casualties of RTI in other African countries were: Kenya 37%^1^, Ethiopia 37%^1^, Ghana 46%^1^ and Cote d’ Ivoire 40%.^1^

Studies have shown lower percentages of RTI pedestrian victims in high-income countries: 11.2% in Vietnam,^11^ 13% in the United States, 12% in Canada, 12% in Malaysia,^12^ 9% in Thailand^13^ and 18% in Hong Kong.^14^ This illustrates that SA follows the African and worldwide low- to middle-income trends in RTIs (Figure 3b).

## Pedestrians as road users in South Africa

Pedestrians still remain the top victim of RTI in SA. The December 2019 festive period RTI mortality showed that the majority of road users who died on the roads were pedestrians (40%), passengers (34%), drivers (25%) and cyclists (1%).^7^ Studies have shown that factors associated with increased risk of pedestrian RTIs include: high vehicle volume, absent lane demarcations, high vehicle speed, high street vendor density and more children living in a given settlement.^15^ The study also identified factors associated with reduction in child pedestrian RTIs as more hours per day spent in school and more years of family residency in the same home.^15^ The family physician has the duty to promote these preventative measures in collaboration with the network of other stakeholders, for example, the law enforcement agents.

## South African road traffic injuries: Where do we stand?

We have not been able to identify any study that describes the spatial and temporal pattern of RTI in SA; however, it has been observed that high road accident mortalities across SA correspond with festive seasons. This could be explained from the high urban–rural migrations during these seasons. The regional determinants of RTI in SA are variable. However, they all revolve round the causative factors mentioned above.

South Africa has a good institutional framework for road safety, but there is laxity in the enforcement of certain criteria such as speed limit, alcohol-level limit and national seatbelt laws.^1^ There is neither national child restraint law nor hands-free phones law.^1^

The launch of ‘365-Days Action Agenda’ in October 2019^7^ and the launch of ‘Arrive Alive campaign’ in December 2019 introduced by the Department of Transport are good initiatives that can reduce the overall RTI in SA. Political will to sustain these initiatives is needed to make the SA roads safe for all.

We as family physician should refocus our pattern of clinical reasoning to recognise RTIs as a risk factor of untimely deaths and disabilities. Road safety health promotion should be encouraged and practised at all times. All stakeholders need to cooperate to reduce the disability – adjusted life year (DALYS), year of life loss (YLL) and the National Life lived with disability (YLD) through RTIs for all our patients.

## Recommendations for prevention of road traffic injuries

We make the following recommendations based on the identified risk factors of RTIs in SA:

- Road safety awareness should be introduced from childhood and maintained throughout schooling. The secondary school curriculum should include ‘Driving’ as a compulsory subject that must be examined at the final matriculation examination.
- Television video and computer games that promote speeding in children should be discouraged. Motor sports involving risky riding or driving should be accompanied by a warning that speeding in public roads is a criminal offence (something similar to the warning accompanying the sale of tobacco products).
- All drunk drivers should be investigated for alcohol use disorder and referred for proper management and rehabilitation if necessary.
- The maximum speed limit is currently 120 km/h for all motor vehicles. There is evidence that reducing speed limit is accompanied by a corresponding reduced percentage in RTIs.^16^ Given the high RTIs at the current speed limits, the current speed limit should be reduced to 100 km/h at the national roads and reduced by 10 km/h at the current speed zones on all other roads. Furthermore, electromechanical automatic deceleration technology should be standard for all motor vehicles so as to prevent driving above the set speed limits.
- All roads (local, provincial and national) should be regularly maintained. Footpaths and cycle routes should be mandatory for all roads and zebra crossings clearly demarcated. All road markings and signage should be renewed frequently.
- The Euro NCAP (New Car Assessment Program) should be introduced for all motor vehicles, with the minimum score set above 3 to minimise crash tendencies. Car painting ensuring visibility should be mandatory.
- Traffic camera installation should be at RTI-prone areas. Pedestrians caught on cameras indulging in dangerous practices, for example, wearing ear phones within or across accident prone areas should be charged with an offense.

## Conclusion

In the advent of advancing technology, every living human being is at risk of RTIs. The safest way to avoid becoming an RTI patient is to adhere to road regulations and life style modification for those leading high-risk behaviour. The role of a family physician under the circumstances cannot be under-estimated. Meeting the challenge of RTIs requires a multi-sectorial approach to raise awareness and enforce the prescribed regulations.
